# Retro-Odontoid Calcium Pyrophosphate Deposition Pseudotumor Presenting with Isolated Hypoglossal Nerve Palsy: A Case Report

**DOI:** 10.1055/a-2952-7337

**Published:** 2026-10-09

**Authors:** Hanna Veronika Salvotti, Luca Zavatto, Andrea Prontera, Pier Paolo Berti, Vania Pirillo

**Affiliations:** 1 Neurosurgery Unit 18513 Hospital of Bolzano (Südtiroler Sanitätsbetrieb - Azienda Sanitaria dell' Alto Adige (SABES-ASDAA)), Teaching Hospital of Paracelsus Medical UniversityBolzanoTrentino-Alto Adige/South TyrolItaly

**Keywords:** retro-odontoid pseudotumor, hypoglossal nerve palsy, calcium pyrophosphate deposition disease, posterior C1–C2 fusion

## Abstract

**Introduction** Retro-odontoid pseudotumor is a rare non-neoplastic lesion of the craniocervical junction, most commonly associated with inflammatory or degenerative conditions. Calcium pyrophosphate deposition (CPPD) disease is an underrecognized cause. Cranial nerve involvement is uncommon, and isolated hypoglossal nerve palsy is exceedingly rare.

**Case Report** We report a 75-year-old woman who presented with a 5-month history of occipital headache followed by acute onset of left hypoglossal nerve palsy. Imaging revealed a partially calcified retro-odontoid mass extending toward the left hypoglossal canal. Laboratory and rheumatologic investigations were unremarkable. The patient underwent posterior C1–C2 fusion with biopsy. Histopathology confirmed CPPD disease. At 6-month follow-up, magnetic resonance imaging (MRI) demonstrated significant regression of the lesion accompanied by clinical improvement.

**Conclusion** This case highlights that posterior stabilization alone may provide effective indirect decompression and promote lesion regression while avoiding more invasive anterior approaches.

## Introduction

Calcium pyrophosphate deposition (CPPD) disease is characterized by the accumulation of calcium pyrophosphate dihydrate crystals within articular cartilage, leading to inflammation and joint degeneration. 1 2 Its prevalence increases with age, affecting up to 50% of individuals over 80 years old. 2 CPPD disease most commonly involves larger and weight-bearing peripheral joints; however, involvement of the craniocervical junction is rare. 3 In this region, CPPD disease may lead to the development of a retro-odontoid pseudotumor (ROP), a non-neoplastic soft tissue mass located posterior to the odontoid process. 4 Differential diagnoses include inflammatory arthropathy, infection, neoplasm, and crystal deposition disease. 5 The pathogenesis of ROP is thought to involve chronic inflammation and fibrocartilaginous metaplasia associated with atlantoaxial instability. 5 6 Magnetic resonance imaging (MRI) is considered the gold standard diagnostic technique for the detection of ROP, with T2-weighted sequences being capable of identifying edema and degree of spinal cord compression and precontrast T1 images detecting mineralization and hemorrhage. On computed tomography (CT), the ROP usually presents with mild enhancement with variable mineralization. 5 ROP typically presents with symptoms of cervicomedullary compression, including myelopathy, gait disturbance, and limb weakness. 7 Cranial nerve involvement is uncommon. While abducens nerve palsy has been described, isolated hypoglossal nerve palsy remains exceedingly rare. 8 9 10 Given the anatomical proximity of the hypoglossal canal to the foramen magnum, lateral extension of a retro-odontoid lesion may result in nerve compression. 11 Surgical treatment for ROP is mainly guided by clinical presentation and the presence of atlantoaxial instability and includes ventral or dorsal decompression and single-step fixation procedures. 12 Anterior odontoid decompression, often performed via a transoral or endoscopic endonasal approach, allows direct mass resection and is generally adopted in cases with cord compression. Although effective, this technique is technically demanding and associated with a higher risk of complications. By elucidating this particularly rare case of CPPD disease-caused ROP manifesting with isolated hypoglossal nerve palsy, we aim to underline that indirect decompression through posterior stabilization alone may be sufficient for symptom relief and mass shrinkage. 13

## Case Report

A 75-year-old woman with a history of osteoarthritis, osteopenia, supraspinatus tendinopathy, asthma, and hypertension presented with a 5-month history of occipital headache followed by acute onset of left-sided hypoglossal nerve palsy. Clinical examination revealed dysarthria and dysphagia without evidence of tongue atrophy. CT demonstrated a partially calcified extra-axial lesion at the level of the dens of C2, extending cranially toward the left hypoglossal canal ( Fig. 1 ). Degenerative fusion of the subaxial cervical spine (C3–C5) was also observed. MRI confirmed a retro-odontoid lesion measuring approximately 42 mm in the sagittal plane and 29 mm in the axial plane. The lesion showed peripheral contrast enhancement on T1-weighted postcontrast sequences and low-to-intermediate signal intensity on T2-weighted imaging, suggestive of mineralized tissue ( Fig. 2 ). 14 Rheumatologic evaluation was unremarkable, and inflammatory markers were within normal limits. Whole-body imaging excluded malignancy. The presence of periodontoid calcifications strongly supported the diagnosis of CPPD disease. 15 The patient underwent posterior C1–C2 fusion with intraoperative biopsy ( Fig. 3 ). Histopathological analysis demonstrated fibrous stromal tissue with birefringent crystal deposits under polarized light, consistent with CPPD ( Fig. 4 ). Postoperatively, colchicine therapy was initiated. At 6-month follow-up, MRI demonstrated marked regression of the lesion ( Fig. 5 ). Clinical recovery occurred progressively over the postoperative course. Dysphagia showed substantial improvement by 3 months after surgery, followed by marked improvement in dysarthria by 6 months. Improvement of tongue atrophy was observed at the 9-month follow-up.

**Fig. 1:**
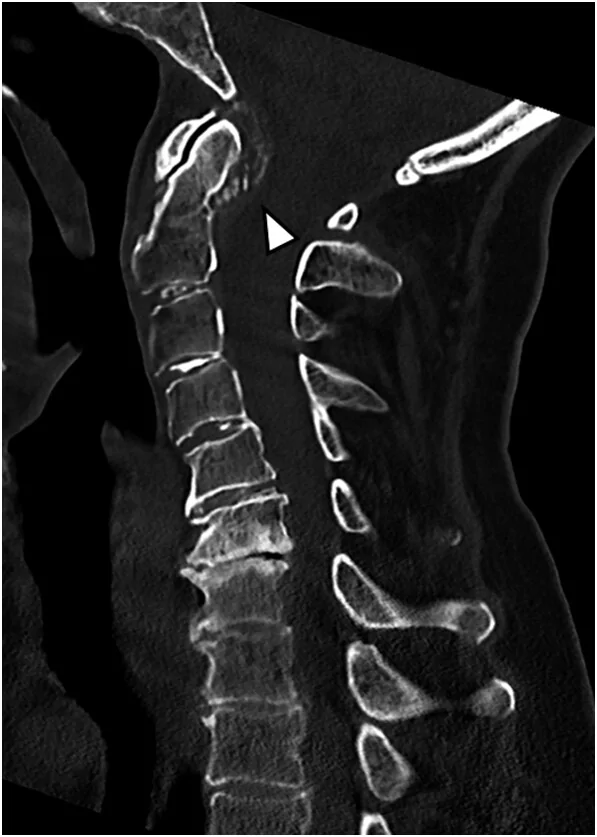
Preoperative CT findings. Sagittal computed tomography (CT) image demonstrating a partially calcified retro-odontoid pseudotumor posterior to the dens of C2, with prominent periodontoid calcifications consistent with calcium pyrophosphate deposition disease.

**Fig. 2:**
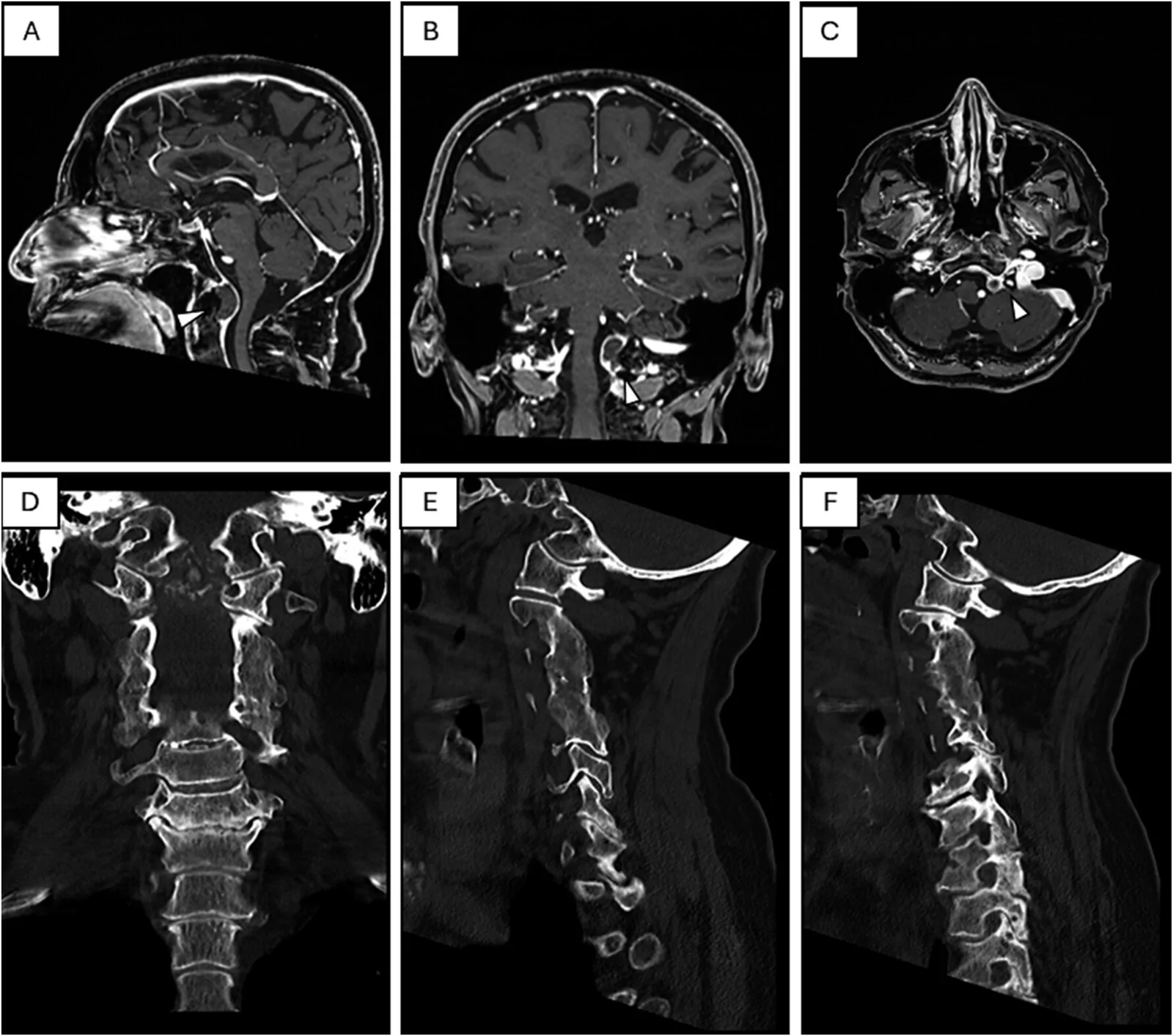
Preoperative MRI findings. Preoperative magnetic resonance imaging (MRI) demonstrating the extent of the retro-odontoid pseudotumor within the spinal canal on sagittal ( **A** ) and coronal ( **B** ) sequences. The axial image ( **C** ) shows displacement of the left hypoglossal nerve by the mass. Preoperative computed tomography images ( **D–F** ) demonstrate fusion of the subaxial cervical spine.

**Fig. 3:**
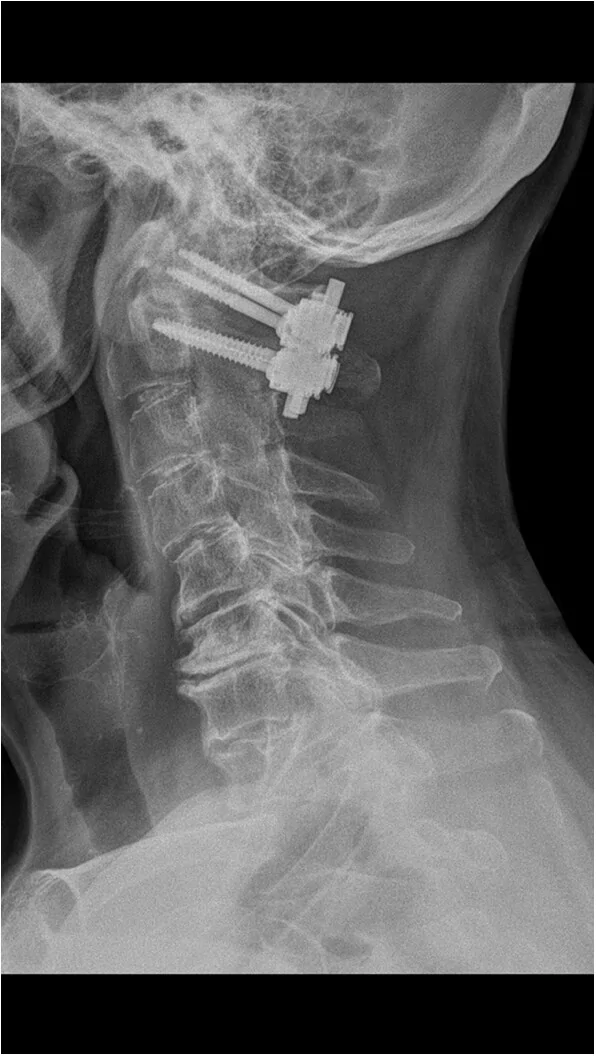
Postoperative radiograph. Postoperative cervical spine radiograph demonstrating C1–C2 fusion performed using the Goel–Harms technique.

**Fig. 4:**
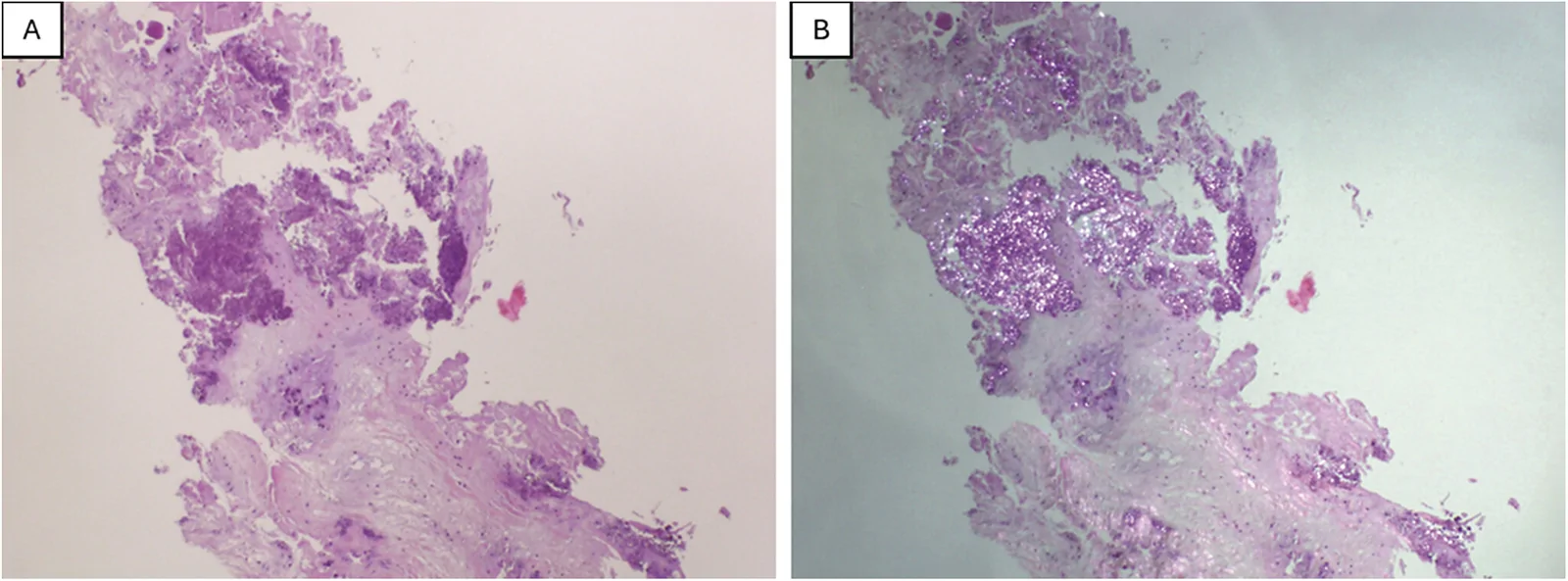
Histopathological examination. Histopathological specimen showing sections stained with hematoxylin and eosin (H&E) of stromal tissue with deposition of amorphous crystals birefringent under polarized light, consistent with calcium pyrophosphate deposits ( **A, B** ).

**Fig. 5:**
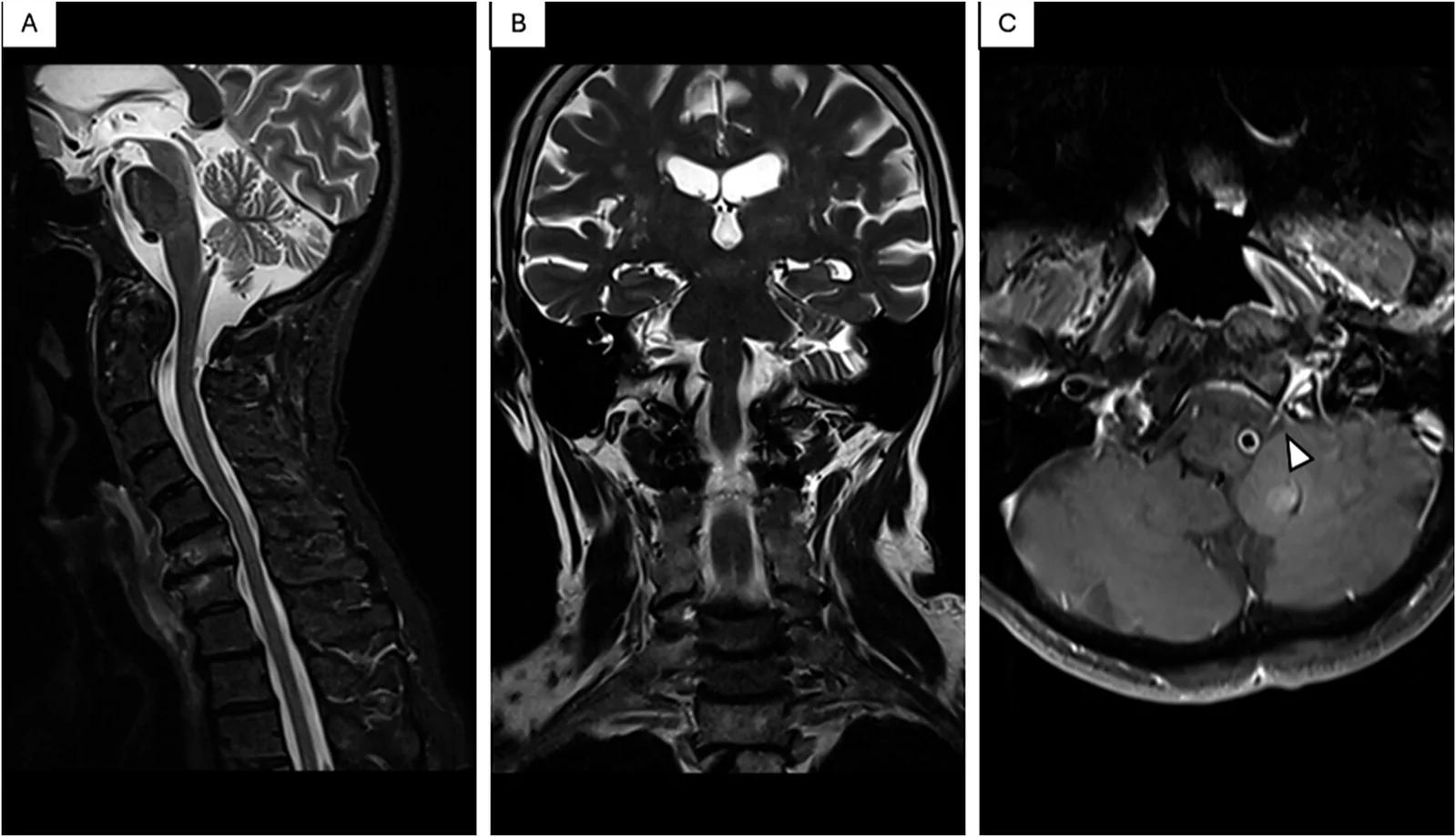
Postoperative MRI at 6-month follow-up. Magnetic resonance imaging (MRI) performed 6 months after surgery showed a marked reduction in the size of the retro-odontoid pseudotumor on sagittal T2-short tau inversion recovery ( **A** ) and coronal T2-weighted ( **B** ) sequences. The axial T1-weighted postcontrast image ( **C** ) demonstrates resolution of the mass effect and decompression of the left hypoglossal nerve.

## Discussion

ROP represents a heterogeneous group of non-neoplastic lesions of the craniocervical junction, most commonly associated with rheumatoid arthritis but increasingly recognized in CPPD disease. 5 16 The pathophysiology of CPPD-related ROP involves chronic crystal deposition leading to persistent inflammation and foreign body granulomatous reaction. 1 6 Atlantoaxial instability plays a central role by promoting repetitive microtrauma and mechanical stress on the transverse ligament. 17 In our case, degenerative fusion of the subaxial cervical spine likely increased biomechanical load at the C1–C2 level. ROP most commonly presents with myelopathy due to spinal cord compression. 7 Isolated cranial nerve dysfunction is rare. Weindling et al highlighted that hypoglossal nerve palsy secondary to craniocervical junction degenerative disease may be underrecognized. 10 A recent case report by Sardi et al described isolated abducens nerve palsy associated with ROP exhibiting superolateral extension, further highlighting the potential of these lesions to involve cranial nerves. 8 The most common causes of acute hypoglossal nerve palsy include demyelinating disorders, ischemic infarction, and intracranial neoplasms or hemorrhage. 18 However, given the close anatomical relationship between the hypoglossal canal and the foramen magnum, as well as the potential for compression by retro-odontoid lesions, the craniocervical junction should be carefully evaluated in cases of idiopathic lower cranial nerve palsy. MRI is considered the gold standard for assessing spinal cord compression and soft tissue lesions. In the present case, the lesion demonstrated hypointensity on T2-weighted sequences with peripheral contrast enhancement, suggestive of mineralized tissue. CT further confirmed the presence of calcific material, consistent with CPPD disease. A recent report by Malhotra et al emphasized the importance of combining MRI and CT in the evaluation of craniocervical junction ROPs to achieve an accurate diagnosis. 15 In contrast to the case described by Molina-Gil et al, in which transient clinical and radiological improvement of craniocervical CPPD disease was achieved with conservative management using non-steroidal anti-inflammatory drugs, our approach focused on addressing the atlantoaxial instability through surgical stabilization. An additional advantage of the surgical strategy is the ability to obtain tissue for histopathological examination, thereby further supporting the diagnosis and strengthening the differential diagnostic process. 9 Surgical treatment is indicated in symptomatic patients or when instability is present. Available approaches include anterior or posterior decompression, combined with posterior stabilization, or posterior stabilization alone. 12 Although anterior approaches allow direct resection and are indispensable when the mass fails to shrink after stabilization or produces severe acute neurological compression, they are associated with higher morbidity. Posterior C1–C2 fusion addresses the underlying instability and may lead to indirect decompression. 12 13 The pathophysiological mechanism behind it is that C1–C2 fixation may promote regression of an ROP by eliminating the pathological micromotion and mechanical stress at the atlantoaxial joint that contribute to its formation, persistence, and growth over time. Once this mechanical stimulus is eliminated, the reactive fibroinflammatory tissue may undergo progressive remodeling and resorption, resulting in gradual reduction of the mass without direct surgical excision. Increasing evidence indicates that stabilization alone can result in spontaneous regression of the pseudotumor. 15 This mechanism is illustrated by Niwa et al who demonstrated significant time-dependent regression of non-rheumatoid ROPs following posterior fixation, with most of the reduction occurring within the first 12 months after surgery. 19 Our case supports this concept, demonstrating significant radiological and clinical improvement without direct mass resection.

## Conclusion

To our knowledge, isolated hypoglossal nerve palsy due to CPPD-related ROP has been only rarely reported. This case supports the concept that posterior C1–C2 stabilization alone may be sufficient, even in the presence of cranial nerve compression, by eliminating pathological motion and allowing spontaneous regression of inflammatory tissue, avoiding more invasive anterior procedures.

## Declaration of GenAI Use

During the preparation of this manuscript, the authors used Generative AI to assist with language editing and improvement of readability. The authors reviewed and edited all AI-assisted content and take full responsibility for the final content of the manuscript.
